# Recent Advances in Indoor Localization via Visible Lights: A Survey

**DOI:** 10.3390/s20051382

**Published:** 2020-03-03

**Authors:** A B M Mohaimenur Rahman, Ting Li, Yu Wang

**Affiliations:** 1 Department of Computer Science, University of North Carolina at Charlotte, Charlotte, NC 28223, USA; 2 Division of Natural Science and Mathematics, Oxford College of Emory University, Oxford, GA 30054, USA; tli41@emory.edu; 3 Department of Computer and Information Sciences, Temple University, Philadelphia, PA 19122, USA

**Keywords:** indoor localization, visible light localization, visible light communication

## Abstract

Because of the limitations of the Global Positioning System (GPS) in indoor scenarios, various types of indoor positioning or localization technologies have been proposed and deployed. Wireless radio signals have been widely used for both communication and localization purposes due to their popular availability in indoor spaces. However, the accuracy of indoor localization based purely on radio signals is still not perfect. Recently, visible light communication (VLC) has made use of electromagnetic radiation from light sources for transmitting data. The potential for deploying visible light communication for indoor localization has been investigated in recent years. Visible-light-based localization enjoys low deployment cost, high throughput, and high security. In this article, the most recent advances in visible-light-based indoor localization systems have been reviewed. We strongly believe that visible-light-based localization will become a low-cost and feasible complementary solution for indoor localization and other smart building applications.

## 1. Introduction

Localization has always been an important topic in the history of technology and humanity. It can go back to the early localization techniques for ocean navigation, such as Polynesian navigation, which was used for thousands of years to make long voyages across thousands of miles of the open Pacific Ocean. Modern localization relies primarily on positions determined electronically by receivers collecting information from satellites or base-stations. In recent decades, location-based services (LBS) [1] have helped mankind in various ways and become a part and parcel of people’s lives. We have seen the use of Global Positioning System (GPS) or other localization technologies [2,3,4] in determining a position everywhere, for example, getting from one location to another using navigation tools, tracking an object or personal motions, and creating real-time maps of the world. We have found its implementations in vehicles, portable devices, mining, aviation, military, and so many other areas. Every day, more and more new usages and applications of localization are being invented.

While GPS is the most dominated localization technology in the world, it is also well known that GPS does not work well or is inaccurate in indoor spaces. It is mainly because the signals transmitted from the satellite gets obstructed by the indoor boundaries like walls or ceilings of the buildings. Therefore, alternative indoor localization techniques have to be invented and developed. Recently, there have been tremendous efforts on the research of indoor localization using various wireless medium or communications, for example, using WiFi signals [5,6,7,8,9,10], Radio Frequency Identification (RFID) [11,12,13,14], Bluetooth [15,16,17], Ultra-Wideband (UWB) [18,19], Acoustic Signals [20,21,22], Inertial Sensor/Measurement Unit (IMU) [23,24,25], or other wireless communication techniques. WiFi and Bluetooth based systems have been deployed in real applications more than the others in the last few decades.

Though these indoor localization technologies are able to localize, they might still have certain weaknesses, such as security issues, high power consumption, and low throughput. To make indoor localization systems more feasible and accurate in large-scale and diverse environments, the solution via visible lights becomes a new low-cost complementary to the existing indoor localization methods. Visible lights have been used for visible light communication (VLC) [26], which makes use of the electromagnetic radiation for transmitting data. The potential for deploying visible light communication for indoor localization has been investigated in recent years, and it enjoys low deployment cost, high throughput, and high security. In this article, we review the most recent advances in visible-light-based indoor localization systems. We strongly believe that visible light localization will play an important role in the future indoor localization systems and other smart building applications.

**Figure 1:**
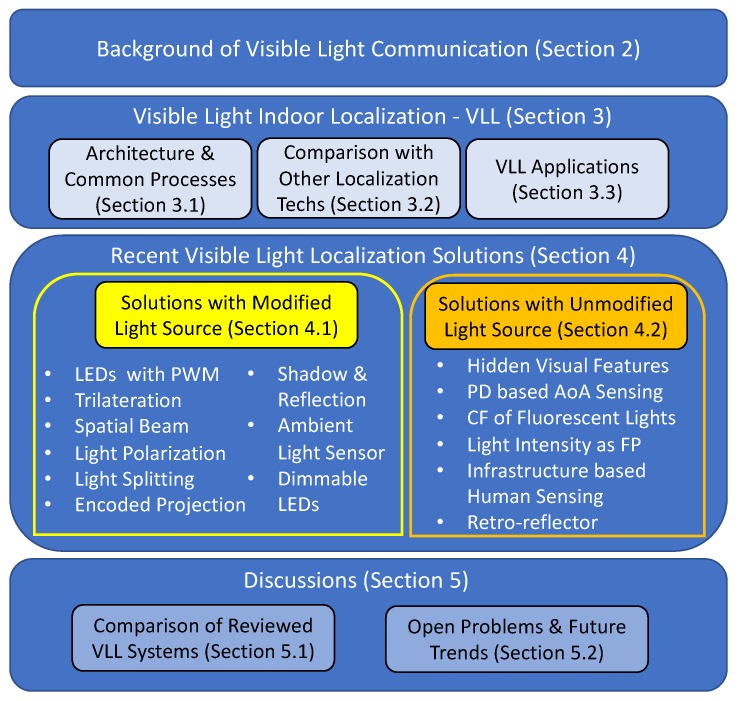
Overview of visible light localization and paper organization.

The overview of this article is shown in Figure 1. Section 2 provides the background on visible light communication, which provides the bases of VLL systems. Section 3 presents a common architecture of indoor localization systems based on visible light, compares it with other indoor localization methods, and discusses its potential applications. Then, detailed reviews on recent advances on visible light indoor localization are provided in Section 4, where all solutions are categorized into two groups based on whether the light sources are modified. Section 5 summarizes all the reviewed methods and discusses potential future research directions. Finally, Section 6 concludes the article with a brief summary.

## 2. Background on Visible Light Communication

In the electromagnetic spectrum, the range in which the rays are perceptible to human eyes (between 400 and 800 THz or 375–780 nm) is generally considered as the *visible light*. Visible light communication [26] is a data communications variant that uses visible light to transmit data wirelessly, and it serves the purpose of both illumination and communication simultaneously. In most VLC, LEDs are used as the light sources, where photo-diodes receive signals from. Compared with traditional wireless communication via radio signals, VLC can achieve a relatively higher throughput, provide a secured way to propagate, with a lower power consumption and a longer life time, as summarized below.

- *Low cost:* LED photo-diodes are very cheap, ranging from less than a dollar to $3, while LED light bulbs are also much cheaper than fluorescent lights.
- *High bandwidth:* Recent efforts in VLC have been focusing on increasing the transmission bandwidth. In 2014, Tsonev et al. [27] presented a gallium nitride LED system, which could achieve the data rate of 3 GB/s.
- *Low power consumption:* LEDs are very power efficient light sources and, thus, an eco-friendly technology. Now, most of the consumers are switching to LEDs from fluorescent bulbs as LEDs give the same brightness for a cheaper price. If all the lights of the world could be replaced by LEDs, then the overall power consumption of the whole world would reduce drastically.
- *High longevity:* LEDs can live up to 10 years with a satisfactory amount of lighting [28].

These promising features of VLC have inspired not only the academia but also the industry and led to many emerging applications. Note that VLC or visible-light-based indoor localization systems can also use other illuminating devices beyond LEDs as their sources, and we will see a few examples in Section 4.

Though there are many advantages of VLC, there are still some challenges that need to be tackled while implementing it in real-world environments. We now briefly list a few of them in Table 1. Note that many of these challenges exist in localization systems that use visible light as well.

## 3. Visible Light Indoor Localization

In recent years, indoor localization techniques based on visible light communication have been proposed and developed [38,39,40]. Laser-based localization methods [41] can also be considered as visible light localization systems, however, they are out of the scope of this survey due to different methodology and application scenarios. In this section, we briefly discuss the common architecture and processes of visible light localization (VLL) systems, compare such systems with other indoor localization technologies, and provide examples of VLL applications.

### 3.1. System Architecture and Common Processes

**Figure 2:**
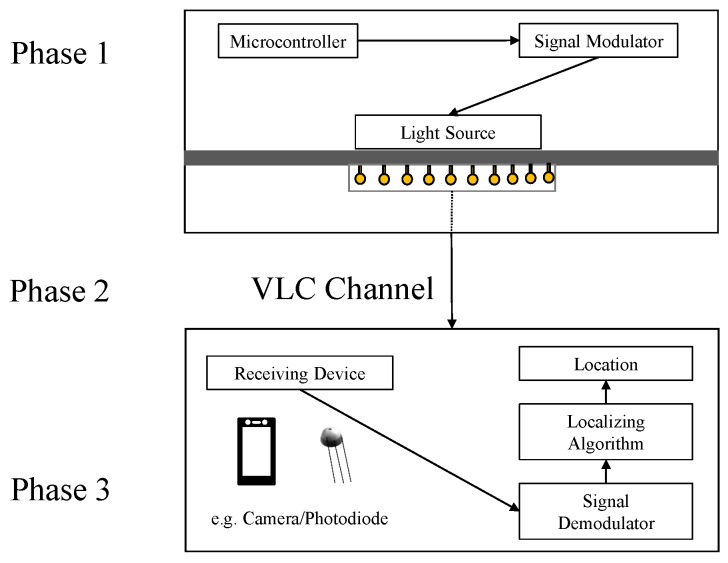
A general architecture for a simplex VLL system.

The visible light localization (VLL) systems generally have two parts in their architecture: *transmitter* and *receiving device*, as shown in Figure 2.

- *Transmitter*. In the transmitter, there is usually a *microcontroller* which controls the *signal modulator* to send certain signals to the *light source* (such as LED array) so that the light source can change its output. In some of the VLL systems, the transmitter part is simply the light source without any modification, while in the others, more complex control and signals are used for the modified light sources.
- *Receiving Device*. The receiving side generally has a *photo-diode/camera* to receive the light signal from the transmitter. The received signal is then passed to the *signal demodulator*. The *localization algorithm* uses the demodulated signal to find out the location.

The localization process in VLL systems based on VLC can be divided into three phases [40], as shown in Figure 2.

- *Phase 1*. A packet data is first encoded into a binary sequence (which is a high–low voltage to control the intensity of light by on–off switching, also known as ON–OFF KEYING (OOK)). OOK is an intensity modulation, which is a prevalent method in VLC. More complex modulation can also be used.
- *Phase 2*. Line of Sight (LoS) paths are required from the light source to the receiver to transmit the data via VLC channels; otherwise, the system may suffer from degradation of signals resulting in a huge amount of inaccuracy.
- *Phase 3*. The final phase of the process is to find out the location of the receiver. The receiver receives the signal and then extracts its characteristics, which are required for the input of the localization algorithm. Some of the examples of these characteristics are Angle of Arrival (AoA), Received Signal Strength (RSS), and Time of Arrival (ToA). The receiver’s location could be known by running the algorithms on the extracted characteristics at the localization module.

**Figure 3:**
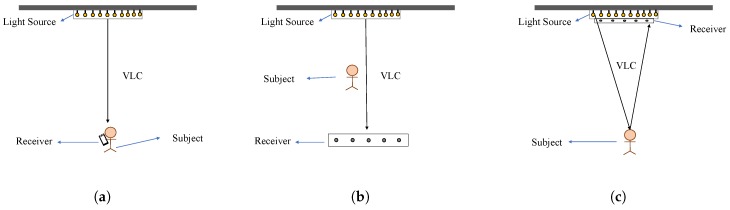
Possible scenarios of VLL systems (**a**) localization of a subject with the receiver; (**b**) localization of a subject between transmitter and receiver; (**c**) localization of a subject with reflection.

The application scenarios of VLL can also be different (beyond the localization of the receiver). Figure 3 shows three possible scenarios.

- *Subject is the receiver*. This is the most common VLL setting, where the subject of localization is the receiver. For example, as shown in Figure 3a, the mobile user holds his smartphone, in which the camera acts as the receiver, and the location of the smartphone is calculated by the VLL system. Such a setting (or a similar setting where a photo-diode is attached to the subject instead of the smartphone’s camera) has been used in [42,43,44,45,46,47,48,49,50,51,52].
- *Subject between the transmitter and receiver*. In this case, the subject of localization is not the receiver. Instead, the subject is moving between the light source and the receiver (see Figure 3b). Such examples of this setting can be found in [53,54].
- *Subject localized via reflection*. Similar to the second scenario, the subject is not the receiver here since the light source and receiver are on the same side of the system (such as both are on the ceiling in Figure 3c). Thus, the localization is done by analyzing the reflection of light (or shadow on the roof). Examples of this setting are [55,56].

### 3.2. Comparison against Other Wireless Localization Technologies

Different indoor localization systems based on various wireless technologies have been proposed and implemented in real-world applications. Compared with other existing wireless techniques (such as WiFi, UWB, or Bluetooth) for indoor localization systems, the accuracy of visible light localization (VLL) systems is usually higher. Some of them have achieved millimeter level accuracy. Note that the accuracy levels in Table 2 are reported from the literature, such as [57]. However, the accuracy of different localization systems heavily depends on the system configuration and deployed environments. For example, localization accuracy based on WiFi or Bluetooth ranges from cm level to *m* level in different systems. Here, we report the best we found in the literature. Different wireless techniques have different transmission ranges [58], and visible light has a relatively longer range than those of UWB, Bluetooth, and acoustic signals but could not pass through walls. VLL also enjoys the lower power consumption and better security. A comparison among all the wireless techniques for indoor localization is summarized in Table 2.

### 3.3. Applications of Visible Light Localization

VLL systems can enable many practical applications and play essential roles in modern life. They can be categorized into three broad areas as follows.

1. **Navigation:** Obviously, VLL systems can be used for indoor navigation and also support location-based services (LBS) in different indoor environments. For example, spaces like theater, museums, and stadiums are places where people might easily get lost and they need indoor localization in order to guide them to their seats or location they want. The staff might also need location services so that they can control the number of visitors arriving. The interesting fact is that these places are already filled with luminaries. So, VLL systems can be easily deployed with just some additional equipment. VLL systems can be installed in shopping centers (which generally have a complicated floor plan with many stalls) to ease the life of the shoppers as well as the sellers/merchants. The merchants can advertise their stalls in an organized way to certainly interested shoppers via LBS. It can also promote personalized shopping experiences by delivering the prices of products and deals going on when the visitor visits a stall. Moreover, VLL systems can also be used in airports and train stations because these are generally very crowded and large spaced. With VLL, the passengers can find the correct routes, train or bus exits, restrooms, toilets, and stores.
2. **Tracking:** VLL can also be used for tracking objects (such as humans, devices, robots, gestures) in indoor environments. In some industries, it is required to locate the staff, products, and assets in an efficient manner. VLL system can be used for tracking of these subjects. Robots can also use VLL to track and manage inventory storage. In airports, the ability to track the luggage via VLL is promising. In health care facilities, VLL can be used to track patients, wheelchairs, or any other medical devices. Emergency services can be made more accessible with effective tracking. Last, VLL-based tracking can also be used as a complementary human–computer interface (such as palm or finger tracking via VLL over a desk, or body gesture recognition in a room).
3. **Security:** In the case of security and safety applications, most of the systems generally require device-free passive localization techniques [59]. VLL systems, as shown in Figure 3b,c, can provide device-free passive indoor localization. Such a system can be developed to detect and track intruders in a wireless environment. Note that traditional security systems, like motion detection or video surveillance, can achieve device-free passive localization. However, VLL can provide complimentary solutions with lower deployment costs and better privacy protection.

## 4. Recent Visible Light Localization Solutions

Indoor localization techniques based on visible light have different solutions, as stated in recent surveys [38,39,40]. New mechanisms based on various designs have been proposed even after these surveys were published. Therefore, in this section, we will provide the most recent advances in this area by reviewing the new solutions of VLL systems. Since a VLL system always have at least a light source and a receiving device and both are crucial components of the system, we can categorize all solutions of VLL systems from either side. In this section, we group all reviewed VLL systems into two main categories: Modified Light Source (Section 4.1) and Unmodified Light Source (Section 4.2), depending on whether the light source is modified or not. On the other hand, the type of receiving device can also be another critical design factor in VLL systems [26]. Generally, there are two types of receivers in VLL systems: photo-detector (or called photo sensor, such as photo-diodes) and imaging sensor (or camera sensor, such as smartphone camera). So we can also classify VLL systems into photo-detector-based and imaging-sensor-based. Though we do not use this classification in this section, the type of receiver in each of the reviewed VLL systems is presented in Table 3.

### 4.1. Solutions with Modified Light Source

In this subsection, we review seven different VLL solutions where the light source is carefully designed and modified for VLL purposes. Such solutions usually lead to better performance but with higher cost of implementation.

#### 4.1.1. LEDs with Pulse Width Modulation

**Figure 4:**
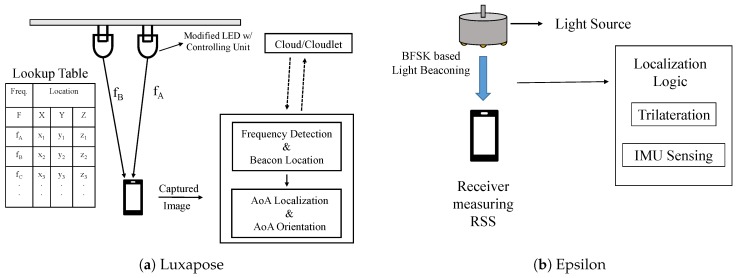
Architectures of (**a**) Luxapose [42] and (**b**) Epsilon [43].

Luxapose [42] makes use of the unmodified phones and slightly modified commercial LED luminaries. Each LED is modified to transmit certain frequencies being fixed with Pulse Width Modulation. The smartphone camera after capturing the image of the luminaries can process the image to decode the transmitted identifiers and use that to find the location of the phone. As shown in Figure 4a, the system mainly consists of three parts: the visible light beacons, a mobile phone, and a cloud/cloudlet server. The visible light beacons are a certain identifier frequency of the LEDs that are imperceptible to humans but captured by the smartphone cameras. To determine the precise location, that information along with the local and cloud resources are used to solve the problem using an angle of arrival localization algorithm. Another similar work where each LED sends localized ID information is [60] by Peng et al. The LEDs in [60] are modulated in Code Division Multiple Access (CDMA). The receiver is a photo-diode, which detects optical signals with ID information from different LEDs. Then, the Tabu search algorithm, a powerful global optimization algorithm, is used to locate the photo-diode.

#### 4.1.2. Trilateration and Fusion of RSS and IMU

Epsilon [43] mainly applies different localization techniques based on the number of available light sources. With three or more light sources, a trilateration/multilateration technique is used based on the Received Signal Strength (RSS) range. With less number of light sources, it will involve the user and leverage the fusion of Inertial Measurement Unit (IMU) sensors like accelerometer, magnetometer, and gyroscope along with the RSS. Its architecture is given in Figure 4b.

- *Trilateration*: Trilateration is mainly a process from geometry where a point is located on the basis of the intersecting shapes, mainly circles. In this case, it is the circular area of the strength received from a certain light source. If the distance from the sources can be calculated precisely, then the intersecting location can be measured from them. The more accurate the measurement of distance is, the more accurate the trilateration.
  The transmitted energy at the light source is a function of the duty cycle of the Pulse Width Modulation (PWM). The light source also needs to deliver the duty cycle information through the beacon for the receiver to correctly model the transmitted power. In Epsilon [43], the RSS measured at the receiver end is calculated as the following equation:
  (1) $$P_{r}=C\sin\left(\frac{\tau }{T}\pi \right)\frac{\cos\theta \cdot \cos\phi }{d^{2}},$$
  where *C* is a constant related to the maximum emission power, and τ/T is the duty cycle of the LED. These two are included in the beacon. θ is the incidence angle, and ϕ is the irradiation angle. And finally, *d* is the actual sender–receiver distance. Epsilon uses Binary Frequency Shift Keying (BFSK) modulation scheme for its beaconing. For localization, it uses Equation (1) to estimate the distance and then computes the 3D coordinates of the receiver uniquely when the number of light sources is four or more.
  Note that trilateration has been widely used in localization systems (including VLL systems). Mousa et al. [61] also proposed a localization system using signal strength-based trilateration. It considers both scenarios of traditional Line of Sight (LoS) and Line of Sight with Non-line of Sight (LoSNLoS) and the effects of noise. For the LoSNLoS case, the effect of first order reflections is considered. Wu et al. [62] used various geometrical and optical formulae derived from trilateration equations to determine the X and Y coordinates. Each LED is modulated by CDMA format with unique ID information related to its geographical position. The Z coordinate is determined using a modified differential evolution (DE) algorithm. This work converts the whole positioning problem into an optimization problem and then tries to optimize it using the DE algorithm. Trilateration has also been used in [63] and [64], but more precisely, they are based on Phase Difference of Arrival (PDoA) or Time Difference of Arrival (TDoA).
- *Involving the user*: This is the case when there is an insufficient number of light sources, i.e., one or two. For solving this type of scenario, two steps are performed in Epsilon [43]. The first step is similar to finding direction using a compass. The user holds the phone horizontally and then rotates the phone along the *Z*-axis to point at the light source. The second step is to gradually pitch the phone, and in the meantime, the RSS values are also collected while the pitch is being changed. With these two steps, the inertial sensors of the phone are used to find out the irradiation and incidence angles, and the orientation angle is also measured. Finally, all of the measured values are put into a localizing function to find out the location.

#### 4.1.3. Spatial Beams

**Figure 5:**
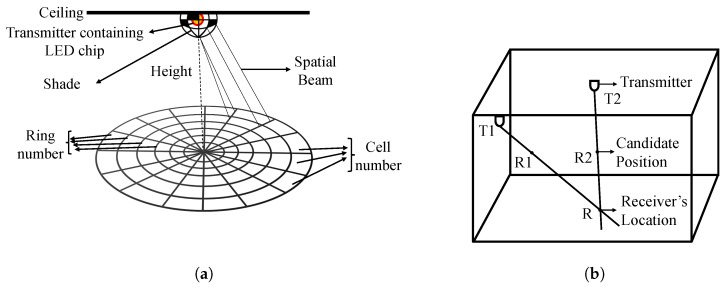
Spinlight [65]: (**a**) Overview of Spinlight with spatial beams and 2D localization; (**b**) 3D localization with two transmitters.

Spinlight [65] uses the idea of spatial beams for the indoor localization process. A light source will transmit spatial beams to a space, and the spatial beams will divide the whole space into specific rings and cells, as shown in Figure 5a. It identifies each beam with a unique timed sequence of light signals. The coded shade that rotates around the LED mainly creates the sequence. Spinlight can perform both 2D and 3D localization.

- *2D Localization*: The whole area of projected space is actually a polar form grid, as shown in Figure 5a. The receiver’s location in polar coordinates would be (r,θ), where *r* is the radius, and θ is the angle. The shade rotates around the LED at a certain rpm, and for the shade to rotate around a cell and again come back to it requires a certain time called the cell period. The controller not only controls the step motor to rotate the shade but also switches on and off the light at two pre-defined frequencies. There is an opto-isolator, which helps the receiver to find its angle. The opto-isolator is a U-shaped object, which transmits infrared (IR) from one side to another. However, there is a plastic barrier in-between so that it blocks the IR light once the cell period triggers a state change, and the controller changes its flash rate. In the meantime, the receiver counts the number of cells passed to estimate its angle. The shade has some hollow parts and some solid parts. The hollow part represents the 1 and the closed solid part represents the 0. So, whenever the shade rotates, the hollow and closed cell comes in turns, and it actually represents a set of bits. So, each ring has a fixed bit pattern in it. And the code contains three parts: (1) leading bits, which helps the receiver to understand the start point of the shade, (2) ring ID bits helps to identify rings and (3) extension bits. The received signals are processed for the cell recognition. The ring ID part gives the receiver’s ring number directly. And the time interval between the first flash rate switch point and the beginning of the leading bits represents the receiver’s cell number. The center of the determined cell is taken as the receiver’s location.
- *3D Localization*: In the case of 3D localization, the received light beam pattern will be the same at different heights. If a line is drawn from the transmitter LED to the receiver, then there are an infinite number of positions or heights that satisfy the same received pattern. However, if there are multiple transmitters, then we can find the intersecting point by drawing lines from them too and find out the exact height of the receiver. In Figure 5b, there are two transmitters $T_{1}$ and $T_{2}$, and the lines drawn from them are $R_{1}$ and $R_{2}$, respectively, which intersect at R to give the height of the receiver.

#### 4.1.4. Light Polarization

We now review three recent VLL systems using polarized light.

**Figure 6:**
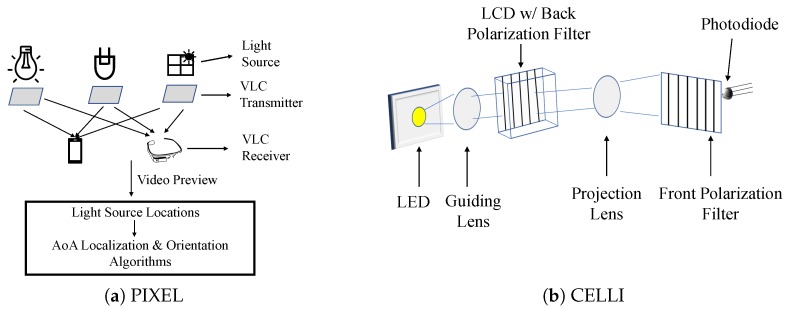
Architectures of (**a**) PIXEL [44] and (**b**) CELLI [68].

**Figure 7:**
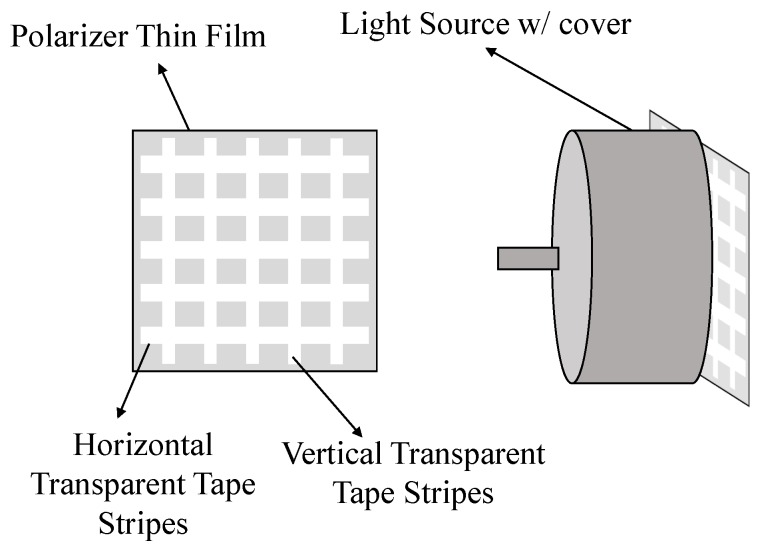
Thin polarizer film with horizontal and vertical transparent tape stripes attached to a light source cover [69].

- *Using Liquid Crystal*: In most of the VLC systems, light flickering is an issue. Modulation is done on the intensity of light, and high rate pulses are needed to transmit. This rate goes beyond 1 kHz so that it is imperceptible to humans. However, for the receiving side, this is a burden. To address this problem, Yang et al. [44] proposed a system, PIXEL, which does modulation on the polarized light via liquid crystal. As shown in Figure 6a, there are mainly three parts of the system: the VLC transmitter, VLC receiver, and the AoA based localization and orientation algorithm. The light source can be any illuminating sources, including the sun light coming through a window. The VLC transmitter is attached to the surface of light sources for polarization. PIXEL is inspired by Liquid Crystal Display (LCD). In LCDs, there are two polarizer layers and one liquid crystal layer in the middle. In PIXEL, the transmitter contains a polarizer layer and dispersor and a liquid crystal in the middle, while the second polarizer layer is on the receiving side. The transmitter implements a modulation scheme known as the Binary Color Shift Keying (BCSK). As the receiving side is a smart phone or wearable device carried by users, there is mobility, which affects the effective intensity difference between the layers. For this reason, PIXEL uses the dispersor so that it splits the polarized light into different colors and causes a difference in the intensity. The receiving smart device captures the beacons using its camera’s video preview. From the video, the relative positions of the light sources can be found. To determine the beacon’s identity, the VLC receiver decodes it with a database that stores the identities corresponding to the light sources. An optimized version of the AoA-based localization and orientation algorithm [42] is applied. The optimization was done by applying the widely used Levenberg–Marquardt algorithm [66]. Inspired from PIXEL, another system called POLI [67] is introduced for visible-light-based communication. In POLI, the optical rotatory dispersor is used to separate the RGB channels and incorporate a point-to-point communication system.
- *Interference-free (IF) Polarized Light Beams*: CELLI by Wei et al. [68] has tweaked the transmitter. A small LCD is installed at the transmitter to project a large number of narrow and interference-free polarized light beams in the spatial domain. These polarized light beams are unique to each projected cell. The receiver then receives the unique transmission and identifies its located cell. As shown in Figure 6b, the guiding lens in front of the LED refracts the light towards the LCD. There is another projection lens to refract the polarized light rays from the LCD to project to the spatial cells. A filter detached from the LCD is attached in front of the receiver. The high spatial resolution of LCD is an advantage that helps CELLI to achieve higher fine-grained positioning. Though the CELLI receiver can calculate the coordinates, it cannot find the absolute location of the receiver. To find out the height’s information, a two-lens strategy at the transmitter side is introduced. Now the receiver receives two values of projection from the transmitter side. The geometrical properties could be leveraged to find the height and the absolute location of the receiver.
- *Light Polarization Pattern with IMU Tracking*: The authors of [69] used ubiquitous lights to correct the errors caused by Inertial Measurement Unit (IMU) tracking and increase the overall localization accuracy. IMU-based tracking methods are widely used but suffer from a famous problem known as the drifting problem. To solve it, many techniques (such as landmark-based and WiFi fingerprints) have been used to correct the drifting errors. The research in [69] cast passive and imperceptible light polarization patterns for the same purpose, and replies on existing indoor luminaries. It attaches a thin polarizer film to the light cover/diffuser to create the polarized light, as shown in Figure 7. This type of polarizer generally allows some kinds of polarized light and blocks. To create a spatial pattern, it makes use of the birefringence property. Transparent tape is used as an anisotropic material, which rotates the polarization of a light ray based on the refractive index using the birefringence property. For this, the white light will be divided into several color light beams in different directions. A colored sensor, covered with a polarizer, monitors its *R*/*G*/*B* channel input for color changes to detect the light pattern and the edge-crossing event.

#### 4.1.5. Light Splitting Properties of Convex Lens

SmartLight [45] exploits the light splitting properties of a convex lens to create a one-to-one mapping between the location and the light rays receivable at that location. This is the first digital approach to achieve 3D localization of multiple objects simultaneously with a single light source. It includes two designs: basic and advanced design.

**Figure 8:**
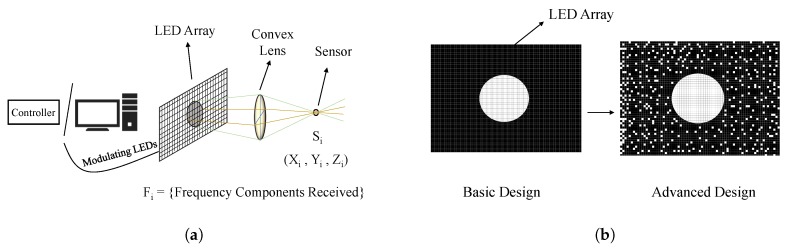
SmartLight [45]: (**a**) Architecture, (**b**) Comparison of bitmap for basic and advanced design.

In the basic design, each of the LEDs in the LED array blinks at a unique frequency. As shown in Figure 8a, the device consists of a square LED array panel placed at the left side of a convex lens and is controlled by the computer (or controller). The sensor, say $S_{i}$, receives a set of frequencies from different LEDs. Then the sensor sends all the frequency components to the SmartLight device as binary decisions. Each sensor has its visible region and also creates a circle area of pixels in the LED array. In the basic design, they make a bitmap, which contains the values 0 and 1 being marked as black and white when it does not match or matches with the blinking frequency. From the bitmap graph and by using a geometrical optical localization formula, they found out the *X*, *Y*, and *Z*-axis values of the sensor $S_{i}$ as:

(2) $$X_{i}=\frac{hfR(N-2X_{c}-D)}{2RN(d_{o}-f)+fhD},\;\;Y_{i}=\frac{hfR(N-2Y_{c}-D)}{2RN(d_{o}-f)+fhD},\;\;Z_{i}=\frac{2RNfd_{o}}{2RN(d_{o}-f)+fhD},$$

where *D* is the number of pixels in the diameter of the circle, *R* is the radius of the lens, *N* is the number of pixels in one dimension of the panel, *f* is the focal length of the lens, $d_{o}$ is the distance from the LED panel to the lens, *h* indicates that the LED panel has the dimension *h* (meter)×h (meter), $X_{c}$ is the number of pixels from the center to the left of the panel, $Y_{c}$ is the number of pixels from the center to the right of the panel. However, the basic design had some limitations. It uses unique frequencies for all the LEDs of the array; thus, it can not be deployed for large scale scenarios. Therefore, the advanced design is proposed.

**Figure 9:**
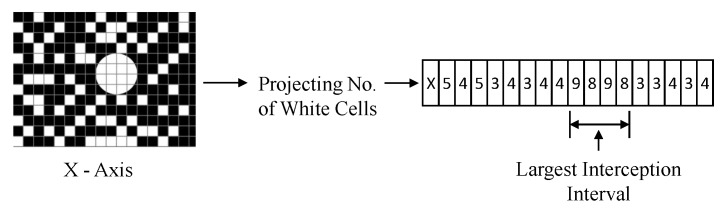
Multi-directional projection for *X*-axis: converting the 2D problem into a 1D problem.

In the advanced design, the frequency of the LED array is reused. When the bitmap is created, there will be a lot of false-positive white dots, as shown in the right figure in Figure 8b. So, now the problem becomes to find out the circular region from this sea of false positives. To solve this problem, a multi-directional projection is used, which plots the aggregated result of each column’s number of white dots on the *X*/*Y* axis, as shown in Figure 9. Then, the 2D problem is converted into a 1D problem of finding the peak. Such a process is done for both the axis. y=ρ∗N is used to intercept the curve where ρ is the probability of a pixel outside the circle area to be marked white, and *N* is the dimension of the LED array.

As different light sensors at different locations receive a different set of light pixels on the LED panel, SmartLight can localize them simultaneously, as it considers each case of sensor separately, and calculate the bitmaps separately to find out the location. Therefore, SmartLight supports parallel localization.

#### 4.1.6. Encoded Projection

**Figure 10:**
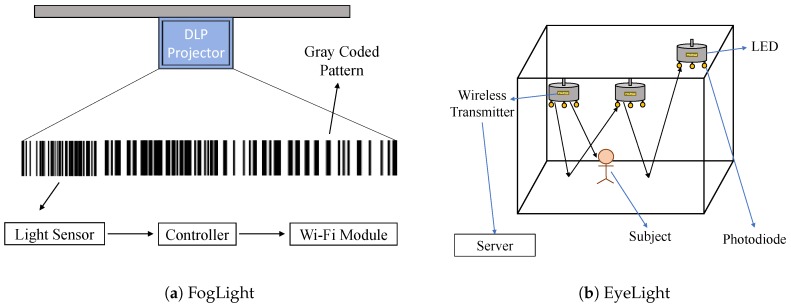
Architectures of (**a**) FogLight [46] and (**b**) EyeLight [55].

Digital Light Processing (DLP) is one kind of projector being commonly used (such as to project the movie on to the screen in movie theaters). The digital micro-mirror device (DMD) chip inside a DLP projector is made up of millions of micro-optical mirrors shaped up like a diamond type pixel array. These mirrors can be alternated between ON and OFF states at a high frequency. This alternating property can be used to modulate light by changing projected images. FogLight [46] exploits this projection property to design a VLL system based on encoded projection. FogLight uses off-the-shelf DLP projectors and light sensors for high-resolution localization, and it leverages the alternating or fast-flipping property of the DLP to project a binary pattern image, which is actually an encode of the projected area. The system architecture of FogLight is shown in Figure 10a. After the light signal is received by the light sensor from the projected gray-coded pattern, it is sent to the controller for decoding and perspective transformation. Then it sends the position via the WiFi module.

FogLight is based on the projection of the encoded projected area using gray-coded binary patterns. Inside the projection area, each pixel is actually a codeword, which is a sequence of binary digits. There is a direct mapping of the codewords to the corresponding pixel coordinates on the projected pattern. FogLight uses two colors (black and white) to represent 0 and 1, respectively. FogLight has embedded synchronization frames inside each data package, so no extra communication channel is needed for synchronization. As the DLP is projecting all the time, so there is an issue of flickering, which may be perceptible to human eyes. FogLight handles the flickering issue by putting reverse bits after each 1 or 0 so that there is always switching between 0 and 1. This makes the projector a stable light source. The sensors are connected to the Arduino Micro-controller using the signal conditioning circuit. Moreover, this circuit contains amplifier and voltage comparator, which increase the signal strength when the sensors are far away from the source and reduce the unwanted small noises from the signals.

#### 4.1.7. Shadow and Reflection

We experience shadows all the time. Even if there is a small amount of light in the space, there will be a shadow. EyeLight [55] integrates photo sensors with existing light bulbs (which reduces the installation and maintenance costs) and exploits the reflections of the light off the surface. As shown in Figure 10b, light bulbs have wireless connectivity to report the readings from the sensors to the server. This server then applies the tracking and activity detection algorithms to process the data to localize the occupants. One key requirement of the design is that the receiver should be able to isolate the light received from different light sources. To satisfy this condition, ON–OFF signaling is used, and it is not perceptible to the human eye. Furthermore, periodic signaling is used so that it does not affect the overall light intensity or illumination level. At the receiver side, photo-diode is used as the sensor since it can detect weak signals and respond quickly. A trans-impedance amplifier is used to amplify the weak current the photo-diode produces. An instrumentation amplifier is also used to boost the gain. To detect each LED, a uniquely time-slot based mechanism is applied.

Two methods are introduced for localization: Spike algorithm, which is coarse-grained, and Delta algorithm, which is fine-grained. Spike algorithm is a simple one, which just detects if there is a person walking by or not. It works mainly with received light power level. It tries to detect the change by taking the average of the received light power continuously and then deciding on the basis of the threshold value, which is determined beforehand when the room or space is empty. Delta algorithm detects the change specifically between a certain pair of transmitter-receiver links. It considers the reflected lights from the surface to the receiver as a virtual light barrier. So, if the barrier is broken, that means the occupant is within that link. It measures the received light power of a sensor when the adjacent light node is in its ON and OFF state. The difference between them can eliminate the ambient light’s power, as it is the same for both the ON and OFF state of the light node. Whenever a person crosses the link between any pair of a node, that person actually either blocks the light or reflects the light. This causes the change or deviation in the normal delta value level, which helps to decide where the occupant is.

Another similar system is STARLIT [70], which uses light reflection for positioning as well. STARLIT only uses one LED to localize by exploiting the rolling shutter mechanism of a smartphone camera, treating it as a sensor array. The LED is modulated by an ON–OFF KEYING (OOK) transistor switch circuit. The different pixel sensors receive the reflected light. The received optical signal strength is different at the different pixels. STARLIT has established a model that relates the received signal strength to the geometric positioning of the LED and the smartphone, which helps to compute the smartphone’s 3D location.

#### 4.1.8. Ambient Light Sensor

**Figure 11:**
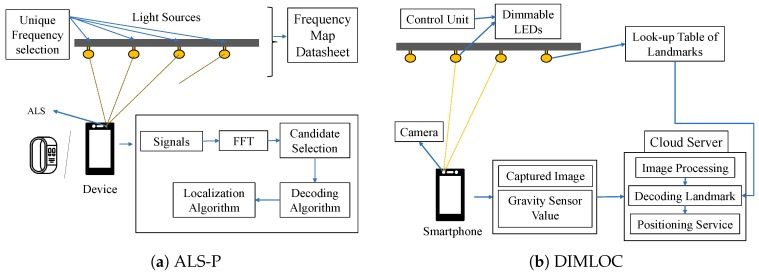
Architectures of (**a**) ALS-P [47] and (**b**) DIMLOC [48].

Most of the systems are not specifically designed for low power devices. Wang et al. [47] recently proposed a lightweight VLL system, ALS-P, which makes use of the under-sampling of Ambient Light Sensor (ALS) to detect the high frequency of LED bulbs. Figure 11a depicts the overview of the system. The modulation rate of an LED bulb is generally higher than 1000 Hz because human eyes are sensitive to low rate changes in light intensity [71]. To receive the high frequency by ALS, ALS-P considers the concept of frequency aliasing effect to sample the input signal with two different sampling rates to differentiate more frequencies. Each LED bulb uses a unique and fixed PWM frequency while transmitting. The frequency selection system generates and stores integral frequencies based on predefined thresholds to handle the impact of the integration effect and the harmonic interference along with keeping the world coordinates of the LEDs. On the receiving side, FFT results of the received signals are passed on to the decoding algorithm to find out the IDs of the LEDs. In the decoding algorithm, candidate frequencies of the LEDs for both sampling rates are selected considering all the combinations and then checking their distance in the real world. If the combination gives a distance less than the predefined threshold, then that selection is finalized. The final step is to localize the device for which a trilateration technique similar to the optical model of [43] is used.

#### 4.1.9. Dimmable LEDs

Most VLL systems do not work under dimmable LEDs because of the blurring effects; thus, Liu et al. [48] proposed DIMLOC, a VLL system for dimmable LEDs. As shown in Figure 11b, DIMLOC includes mainly three components—dimmable LEDs, a smartphone, and a cloud server. The LEDs are the transmitters that transmit a unique landmark to represent its world coordinates. The smartphone receives the transmitted signal by capturing the image of the LEDs in the field of view of the front lens. The captured LEDs in the image have bright and dark stripes that form the stripe information to represent its landmark. The captured image, along with the gravity sensor data from the smartphone’s accelerometer, is passed on to the cloud server for further processing to decode the landmark and determine the world coordinates of the smartphone. In the cloud server, there are mainly two modules—one for decoding landmark using image processing techniques, and the other one for localizing the smartphone based on visual analysis and scaling factor principles. The system considers two scenarios while determining the location of the smartphone. One of the scenarios is where the screen of the smartphone is parallel to the ceiling, and the other is where the smartphone tilts at an angle.

### 4.2. Solutions with Unmodified Light Source

Next, we review the solutions that do not modify the light source. Such solutions lead to smaller implementation costs, but the signal processing techniques and localization algorithms at the receiver are usually more complex than those in the VLL systems with modified light sources.

#### 4.2.1. Hidden Visual Features of Lamps

**Figure 12:**
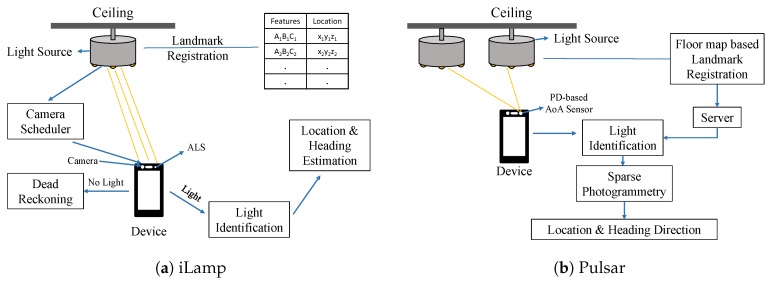
Architectures of (**a**) iLamp [49] and (**b**) Pulsar [50].

Zhu et al. [49] claim that every lamp has its intrinsic characteristics or visual features. They proposed the iLAMP (indoor Light Assisted Mobile Positioning) system, as shown in Figure 12a, which leverages the hidden visual features of lamps to perform localization. It captures images of lamps and processes them using its computation imaging framework to extract the features to identify certain lamps. Moreover, it can estimate the phone’s 3D location by using a geometric model that combines the camera image with the gyroscope and accelerometer readings. The heading direction relative to each lamp landmark could also be found out. iLAMP mainly consists of three main modules: (a) light identification, (2) phone location/heading estimation, and (3) camera scheduling.

The landmarks need to be first registered. Benchmark images of all the lamps are taken, and visual features are extracted from them and registered in the server database. During the localization, the camera captures the image, extracts the main features from the image, which is the spatial radiance pattern, and compress it into an array. In addition, iLAMP finds two more assistant features: colored pattern and the infrared-to-visible-light intensity ratio. A vector is formed with these three features and sent to the server for running the hierarchical light identification algorithm. Such an algorithm can identify the light’s location or landmark. Then, iLAMP makes use of a sensor assisted photogrammetry to calculate the phone’s 3D location with respect to the light landmark based on the phone’s camera image and the gravity sensor output. Moreover, to contain the power consumption, camera scheduling adaptively turns on the camera.

iLAMP mainly uses its light identification module and phone’s sensors to identify the phone’s location when it receives at least one full light. Whenever it does not receive any light, iLAMP uses a motion sensor based dead-reckoning method to keep track of the phone’s movement.

#### 4.2.2. PD-Based AoA Sensing

Pulsar [50] used a compact photo-diode (PD) sensor to differentiate ceiling lights based on their latent optical emission features. PD sensors have higher dynamic range and can capture LED’s internal frequency from a far distance, say 9m away. Pulsar uses a novel mechanism, sparse photogrammetry, to resolve the light source’s angle of arrival and then triangulates the phone’s location and sometimes even orientation based on the number of LEDs in the scenario. Figure 12b shows the architecture of Pulsar.

Sparse photogrammetry derives the Angle of Arrival (AoA) of the light sources based on the compact light sensor. The sensor mainly contains two photo-diodes with different Fields of View (FoV). The differential response between them follows a non-linear function with the AoA. This can be calibrated and known beforehand when it is manufactured. By measuring these responses, it can map them to the light source’s AoA. Pulsar uses AoA in place of RSS to find its way out of the Lambertian model. This enables Pulsar to localize lights of any shape. Using a triangulation model, it can find out the device’s 3D location. If there are more than three lights, the Pulsar sensor can also find out the orientation angles.

Like other VLL systems, Pulsar also needs initial bootstrapping. A surveyor walks inside the building capturing the frequency features of the lights, and the lights’ location gets registered on the publicly available floor map and stored in the database.

#### 4.2.3. Characteristic Frequency of Fluorescent Lights

LiTell [51,72] makes similar observations as [49] but focuses on fluorescent lights that each fluorescent light has its own unique characteristic frequency (CF). The fundamental frequency of FLs is generally within the frequency 40–60 kHz, followed by its integer multiples. The different resonance frequency causes each of the lights to flicker in a different characteristic frequency. LiTell makes use of this distinctive feature. It implements a set of sampling, signal amplification, and camera optimization mechanisms that enable a smartphone camera to capture the weak and high frequency (>80 kHz) features. These frequency ranges are outside human perception.

**Figure 13:**
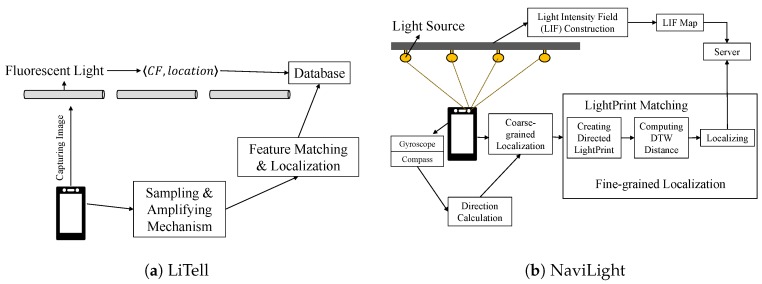
Architectures of (**a**) LiTell [51] and (**b**) NaviLight [52].

The localization technique of LiTell is actually simple. As shown in Figure 13a, it firstly finds out the characteristic frequencies of all the lights and stores them in the database as a fingerprint. Then, when the localization process starts, the smartphone captures the image of the fluorescent light and then runs a sampling and amplifying mechanism to find out the characteristic frequency. By matching the CF with the one in the database, LiTell finds out the location of the phone.

#### 4.2.4. Light Intensity as Fingerprints

NaviLight [52] makes use of light intensity as light fingerprints (called “LightPrint”) of light sources, and it treats unmodified existing light sources as transmitters to detect the location of the user. However, light intensity as a fingerprint is not as easy as the WiFi Received Signal Strength Indicator (RSSI) fingerprint (which is widely used by WiFi-based indoor localization systems). Light intensity is more coarse-grained and ambiguous over space as compared with the electronic signal strength, and the communication channel between the source and the receiver may not exist in the VLL system [52]. Therefore, NaviLight uses a vector of light intensities combined with the user’s walk or movement as the determining factor. Note that just using the light intensity of a location is not sufficient since the light intensity of one location might be similar to another location’s light intensity. Matching the LightPrints with pre-trained data and finding out the position in the light intensity floor (LIF) map can be computationally expensive. NaviLight breaks down into two steps: coarse-grained and fine-grained localization, as shown in Figure 13b.

The coarse-grained part contains the application of k-nearest neighbors (KNN), a very well-known classifier, to localize the sub-area within the whole floor map to make the system more scalable. It does not matter how large the floor map is, KNN is going to find out the smaller sub area and pass it to the next stage for fine-grained localization. As it is collected during the user’s movement, LightPrints are highly likely to be curves in physical space. Therefore, directed LightPrints are complicated to match with the LIF map. NaviLight partitions the large LightPrint vectors into many small sized vectors based on IMU data, and then mapping the corresponding directed and segmented LightPrint against light intensity sequences in the LIF map can be done more efficiently. Since LightPrints vary due to the varying walking speed of the users, NaviLight uses subsequence Dynamic Time Warping (DTW) in the light intensity sequences in the LIF and then matches it with the LightPrints to find out which one has the minimum DTW distance. To deal with the ambiguity caused by LIF regularities, a clustering method based on DTW distances is used to find the best match. The location of the cluster that had the minimum average DTW distance was selected.

Similar solutions have been proposed based on various classification methods over the received signal strengths of lights [73,74,75,76,77,78] recently. Both [73] and [74] also used the KNN classifier for their localization algorithms. The latter one relies on the signal features from the flicker frequency spectra followed by KNN clustering. In [75], multiple classifiers were leveraged, and two fusion localization algorithms were proposed (i.e., grid-independent and grid-dependent least square) to combine the outputs of multiple trained classifiers, whereas [77] used two popular functions (classification and regression) of ML-based algorithms (such as KNN, decision trees (DT), support vector machines (SVM), and random forest (RF)). On the other hand, [76] and [78] leveraged neural networks in their VLL systems. In [76], the three axes of the coordinate of the receiver location based on three neural networks were inferred, while [78] used an artificial neural network with one hidden layer trained by the modified momentum back propagation method. In both solutions, the input to the layers is mainly the RSS data-sets of the transmitting LEDs.

#### 4.2.5. Infra-Structure-based Human Sensing

Starlight [53] makes use of the light emitted from the ceiling LED panels to reconstruct fine grained human skeleton postures continuously in real time. Starlight is a fully light sensing system consisting of LED panels and low cost photo-diodes. It does not use any high end cameras, on-body sensors, and electromagnetic interference. It is purely just light sensors collecting shadow information created by our body blocking the lights and recovering the behavior instantaneously. Thus, it removes the need for high fidelity sensors, and the light-based sensing protects user privacy too. Note that Starlight is different from STARLIT and EyeLight where LEDs are modified with modulation signals.

**Figure 14:**
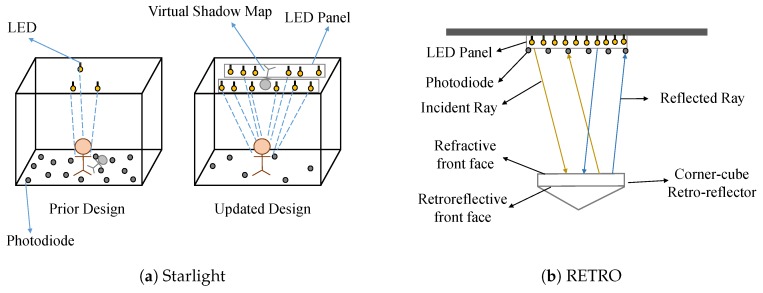
(**a**) Comparison of architectures in Starlight [53]; (**b**) architectures of RETRO [56].

Starlight uses a number of photo-diodes (e.g., 20) on the ground to measure the light blockage data. By leveraging the blockage information of the large number of light rays from the LED panels, it identifies the best fitting 3D skeleton postures. As shown in Figure 14a, the prior design of a similar system had more photo-diodes and less LEDs to collect the shadow information on the floor. To minimize the deployment cost in Starlight, fewer photo-diodes are used with more ceiling LED lights. The new improved architecture of Starlight mainly aims to recover the virtual shadow map projected on the ceiling.

On the other hand, Zhang et al., in their work [79], came up with a reverse technique where rather than using multiple LEDs to locate one receiver, they used multiple PDs on the ceiling and each target object had one LED. Another work [80] also used multiple photo-diodes (including titled and horizontal photo-diodes) but a single LED lamp. A mathematical relationship was established between the received optical power ratio and the receiver’s three dimensional coordinates according to the relationship of the position between the PDs and the tilt angle, azimuth angle, placement angle of the tilted PD, and other parameters to find out the location information.

#### 4.2.6. Retro-Reflector

We are familiar with plane mirrors, which reflect back the light in the same direction if and only if the incidence angle is 0°. However, what if the angle of incidence is not zero. For this case, we use a retro-reflector or Retroflector, which can reflect back the incident light in a path that is parallel to the incoming path but in an opposite direction. And it can do so with a much lower amount of scattering. RETRO [56] uses this physical property of a retro-reflector to build a VLL system.

All previous VLL systems use one direction of a VLC channel from the light source to the receiving side, and there is a lack of real-time backward channels from the device to the light source until RETRO. RETRO can localize passive IoT devices by using a retro-reflector, which does not require any high computation and does the job with minimum latency. As shown in Figure 14b, RETRO uses a corner-cube, retro-reflector, LED panel as a light source and some photo-diodes as the sensor. The retro-reflector reflects the incident ray in a parallel reflected ray, which is then received by the photo-diode. To differentiate between multiple IoT devices, RETRO uses an LCD shutter display on the front face of the retro-reflector to modulate the reflected light. Whenever the device moves and changes locations, the photo-diodes receive different light power or received signal strength. Based on these signal strengths and the trilateration-based localization algorithm, the device can be localized. This VLL system can provide a real-time tracking solution and use any single unmodified light source.

An extension of RETRO is PassiveRETRO [81], which is a completely passive version of the system. It splits the LCD shutter into two parts similar to [44]. So, the tag contains only the bandpass optical filter and the linear polarizer for which it does not require any power supply unit. And in the photo-diode end, there is a linear polarizer, liquid crystal, dispersor, and bandpass optical filter. The optical rotatory dispersor is added mainly to reduce the inter-channel interference.

## 5. Discussions

In this section, we provide some comparative discussions on the reviewed methods and point out some potential future research direction in this area.

### 5.1. Comparison of the Reviewed VLL Systems

We have reviewed several solutions of VLL systems that achieve various accuracy levels of indoor localization using visible light. Each of them has its own advantages and disadvantages, unique experiment settings, and diverse application scenarios. It is not feasible for us to replicate all the experiments in a single environment to compare them, as different VLL systems have dramatically different configurations for their experiments. According to [82], even if the overall configuration of the experiment is the same, variations in LED location will still have an effect on the accuracy of the system. Therefore, here we only summarized the achieved accuracy and the experiment configurations of each method in Table 3, which are reported in each individual work. We also provide information of the methodology used by each method, such as whether the light source is modified, whether a smartphone or a photo sensor (such as photo-diode) is used as the receiver, whether the method is device-free, and whether the system supports localization in 2D or 3D or both. We hope such comparison can provide readers with a rough idea of these methods. For more detail regarding the specific method, please refer to its original paper.

Among all the reviewed methods summarized in Table 3, FogLight [46] has the best reported accuracy. Methods from [49,50,53,56,65,68] also have relatively high accuracy. Usually, the imaging sensor based solution is more accurate than the photo-detector-based one. Although the accuracy of EyeLight [55] is the worst, its concept of using shadows is unique and promising. It demonstrates how shadows or reflections off the surface can be exploited in a device-free visible light localization system. Again, these accuracy rates are heavily dependent on the experimental settings; thus, this comparison is quite limited. It would be nice to see more experimental study or assessment, like [82], conducted by both researchers in the academia and practical system designers in the industry. Some of the solutions involve modification of the light sources or dedicated special equipment, which lead to higher deployment cost. Some of the systems only use smart phones, unmodified LEDs, and cheap PD sensors, which lead to low-cost solutions. Many systems also need intensive data collection (such as fingerprint-based), model training (such as machine-learning-based), or system calibration (such as trilateration-based). The complexity of the VLL system also depends on the number of LEDs or other equipment used and the size of the usage space. There are always tradeoffs among performance, cost, and complexity. Some methods only work for 2D or 3D localization, while others may work for both. The aspect of supporting 2D or 3D localization also has an impact on the accuracy. In most cases, a VLL system for 2D might have better accuracy than its extension to support 3D localization (such as [50,68]) since the over search space is less in 2D than 3D. However, there are also scenarios where 3D localization performs better than 2D, like in the star configuration of [82]. One of the most common disadvantages for all the systems was the Line of Sight (LoS) path requirement. As light can not pass through solid objects, this requirement is a must for any system built based on it.

Compared with results from the most recent surveys [38,39,40], new methods and mechanisms based on various designs have been further proposed. Promising improvements over accuracy and wider application scenarios have been seen. While the field of VLL has progressed (especially in the academia), the VLL technology is not as mature as other indoor localization solutions yet. Similar to VLC, there are still continuous growing commercial interests to VLL solutions/products from many companies in different domains; however, the processes of its commercialization and standardization still require considerable efforts from the industry and standards organizations. Some of the reviewed solutions that do not rely on modified light sources or infrastructures can be easier to be commercialized and deployed.

### 5.2. Open Problems and Future Trends

There has been a lot of progress in the field of VLL systems in recent years, but there still remains open problems or issues that need to be dealt with.

- *Line of Sight (LoS) Problem*. One of the major concerns with all the systems is the line of sight problem. Anything blocking the line of sight between the transmitter and the receiver is halting the whole system or significantly affecting the accuracy. In EyeLight [55], the LoS problem is addressed by leveraging shadows, but the accuracy is not as good as those with LoS. To find a way to solve the LoS problem with better accuracy is still a challenge.
- *Co-existence and Interference*. Another issue is the presence of multiple visible light-based systems in a scenario, which may cause interference to each other. To make a VLL system invulnerable to this type of issue might be another research direction.
- *Integration with Other Sensing/Localization Techniques*. Building new localization systems fusing multiple techniques along with visible light to gain more accuracy is also a promising path for future research. Note that [43,52] have used IMU sensing data to enhance their performances. Wang et al. [83] have exploited the bi-modal magnetic field and ambient light data obtained by smartphone magnetic and light sensors for indoor localization with a deep learning approach based on LSTM (long short-term memory).
- *Advanced Machine Learning*. Recently, advanced machine learning techniques (such as deep learning and reinforcement learning) have made significant impacts in many computer science areas, including smart sensing. However, machine learning techniques have not been widely applied in current VLL systems. There are a few exceptions, for example, KNN is used in [52,73,74], second order regression and polynomial trilateral ML model are used in [84], neural network is used in [76,78,85], and deep LSTM mode is used in [83]. We strongly believe that emerging advanced machine learning techniques can play more important roles in future VLL systems.
- *Device Free*. As most of the systems use devices as the receiver, building a device-free VLL system is still a future research direction. A VLL system without carrying any device (such as [55]) can be applied to a wider range of applications.
- *Mobile Crowd Sensing*. Recently, mobile crowd sensing (MCS) [86,87] has become an emerging sensing paradigm for many mobile sensing applications, including indoor localization [88,89,90,91]. The basic idea is leveraging a large number of mobile users carried with smart devices to collaboratively perform sensing, localization, or tracking tasks. Such an idea can also be used for VLL systems to perform light fingerprint collection or peer-to-peer calibration and may also potentially solve the LoS problem. Recently, in [39], Keskin et al. proposed a cooperative VLL system that leverages the communications among VLC receiver units to improve the accuracy of localization via cooperation. Such system shows the potential of cooperative VLL systems.
- *Security*. Security aspects of VLL systems are still an open research area. Some preliminary discussions have been provided by [92] for VLC, including possible Denial of Service attacks, which use a directional light source to disturb the sink node from receiving a packet via VLC. Note that such attacks can also hurt VLL systems based on VLC. A more thorough study on possible attacks and defenses for VLL systems is critical to wide applications of VLL.
- *Robust Localization*. Last but not least, how to achieve more robust localization is always a challenge. Keskin et al. [39] point out a possible way to achieve robust localization results in the case of mobile entities by using temporal cooperation. Temporal cooperation is to account for the previous steps’ information and use it for the current step. In [93], a two-phase framework is proposed to increase robustness when subject to insufficient anchor lights. The coarse phase produces a weighted proximity estimate with as few as one reference light source within a mobile terminal’s FoV, and then a fine phase performs conventional positioning algorithms if sufficient reference light sources are within the FoV. There is still room for innovation to build a robust system that is more feasible than the existing ones.

## 6. Conclusions

In this article, we briefly reviewed recent advances in indoor localization systems using visible light. It is clear that many important advancements have been made within the last five years to achieve a better accuracy of VLL systems. Different types of techniques (such as spatial beams, polarized light, reflector, light intensity fingerprint, light shadow) have been applied and studied. We strongly believe that further advances in VLL (with new machine learning methods, novel integration of multiple wireless techniques, and stronger security designs) will make indoor localization via visible light more practical and applicable. VLL will not be the sole solution for indoor localization, but it will be one of the most important technologies for future localization and navigation systems, and other smart building applications. 

## Figures and Tables

**Table 1:** Challenges of visible light communication (VLC) (or visible light localization (VLL)) systems.

Challenge	References
Narrow bandwidth modulation of the light source, requiring further development of new modulation and coding techniques	[29,30]
Effects such as shadowing, path-loss, multipath propagation, and background noise effects	[29,31]
Interference with other VLC devices and the ambient light sources	[26,30,31]
Tilt position of the transmitter might cause changes of the transmitted signal	[31,32]
Tilt position of the receiver might cause changes of the received signal	[33]
Multiple access techniques and user mobility issues	[29,30]
Eye safety standards vs. limited transmission distance	[29,34]
Not working in light off mode	[29,31]
Deviations on the LED power due to aging, or tolerances on the power	[35]
Upgrading cost from current infrastructures	[29,36]
Integration with WiFi, Bluetooth, RFID, IMU, and other technologies	[37]

**Table 2:** Comparison among different wireless techniques for indoor localization systems.

Wireless Technique	Transmission Range	Omni- Directional	Interference with	Passes through Opaque Wall	Power Consumption	Range of Accuracy
RFID	Long	Yes	RF Signal	Yes	Low	cm level
Acoustic	Short	Yes	Acoustic	Yes	Medium	cm level
Bluetooth	Short	Yes	RF Signal	Yes	Low	cm level
WiFi	Long	Yes	RF Signal	Yes	Medium	cm level
UWB	Short	Yes	Immune to Interference	Yes	Medium	cm level
Visible Light	Long	No	Light	No	Low	mm level

**Table 3:** Comparison of the majority of reviewed VLL Systems.

VLL Systems	Error (cm)/Percentile	Modified Light Source	Use of Smart Phones	Use of Photo Sensors	Device Free	2D/3D Positioning	Experiment Configurations (Number of LEDs or FLs/Deployed Area (m^2^))	Method Used
Luxapose [42]	10/90%	Yes	Yes	No	No	Both	5 LEDs/0.711×0.737	Phones and Modified LED Luminaries
Epsilon [43]	40/90%	No	Yes	No	No	3D	5 LEDs/5×8 or 2×12 or 3.5×6.5	Trilateration and fusion of RSS and IMU
Spinlight [65]	4/90%	Yes	No	Yes	No	Both	1 LED/circular with radius 5.5 m	Spatial Beams
PIXEL [44]	30/90%	Yes	Yes	No	No	3D	8 LEDs/2.4×1.8	Polarization and Liquid Crystal
CELLI-2D [68]	Median 1.07	Yes	No	Yes	No	2D	1 LED with LCD/height 1.75 m	IF Polarized Light Beams
CELLI-3D [68]	Median 2.65	Yes	No	Yes	No	3D	1 LED with LCD/height 2.25 m	IF Polarized Light Beams
PolarPattern [69]	NA	Yes	No	Yes	No	3D	NA	Light Polarization Pattern
SmartLight [45]	50/90%	Yes	No	Yes	No	3D	76×76 LEDs array/4.4×4 or 4×4	Light Splitting Prop. of Convex Lens
FogLight [46]	0.3/90%	Yes	No	Yes	No	2D	DLP Projector/1.38×0.86	Encoded Projections
EyeLight [55]	250/90%	Yes	No	Yes	Yes	2D	7 LEDs/7.5×6	Shadow
STARLIT [70]	55/80%	Yes	Yes	No	No	3D	1 LED/72 m^2^	Reflection Light
ALS-P [47]	25/90%	Yes	Yes	No	No	3D	4 LEDs/1.5×1.2	Ambient Light Sensor
DIMLOC [48]	9/100%	Yes	Yes	No	No	2D	9 LEDs/3.3×3.15	Dimmable LEDs
iLAMP [49]	3.5/90%	No	Yes	No	No	3D	588 FLs or 190 LEDs+129 FLs or 330 FLs/2.5 m or 3 m or 6 m ceiling	Hidden Visual Features of Lamps
Pulsar-2D [50]	6/90%	No	Yes	Yes	No	2D	64 FLs or 110 CFLs or 157 FLs/3 m or 4 m or 2.8 m ceiling	PD based AoA Sensing
Pulsar-3D [50]	31/90%	No	Yes	Yes	No	3D	64 FLs or 110 CFLs or 157 FLs/3 m or 4 m or 2.8 m ceiling	PD based AoA Sensing
LiTell [51]	10–25/90%	No	Yes	No	No	2D	162 FLs/1000	CF of Fluorescent Lights
NaviLight [52]	35/85%	No	Yes	No	No	2D	130 or 38 or 30 LEDs/625 or 148 or 260	Light Intensity as Fingerprint
Starlight [53]	9/90%	No	No	Yes	No	3D	20 LED Panels/3.6×4.8	Infrastructure based Sensing
RETRO [56]	2/90%	No	No	Yes	No	Both	1 LED Panel/height 1.5 m	Retro-reflector
Assessment [82]	21.1–277.8/95%	Yes	No	Yes	No	Both	4 LEDs (Star or Square)/4 × 4	RSS based Trilateration
